# Genetics of hemostasis: from bedside to bench and back again

**DOI:** 10.1172/JCI183500

**Published:** 2024-11-15

**Authors:** David Ginsburg

**Affiliations:** University of Michigan, Ann Arbor, Michigan, USA.

## The importance of training, mentors, and luck

**Figure 1:**
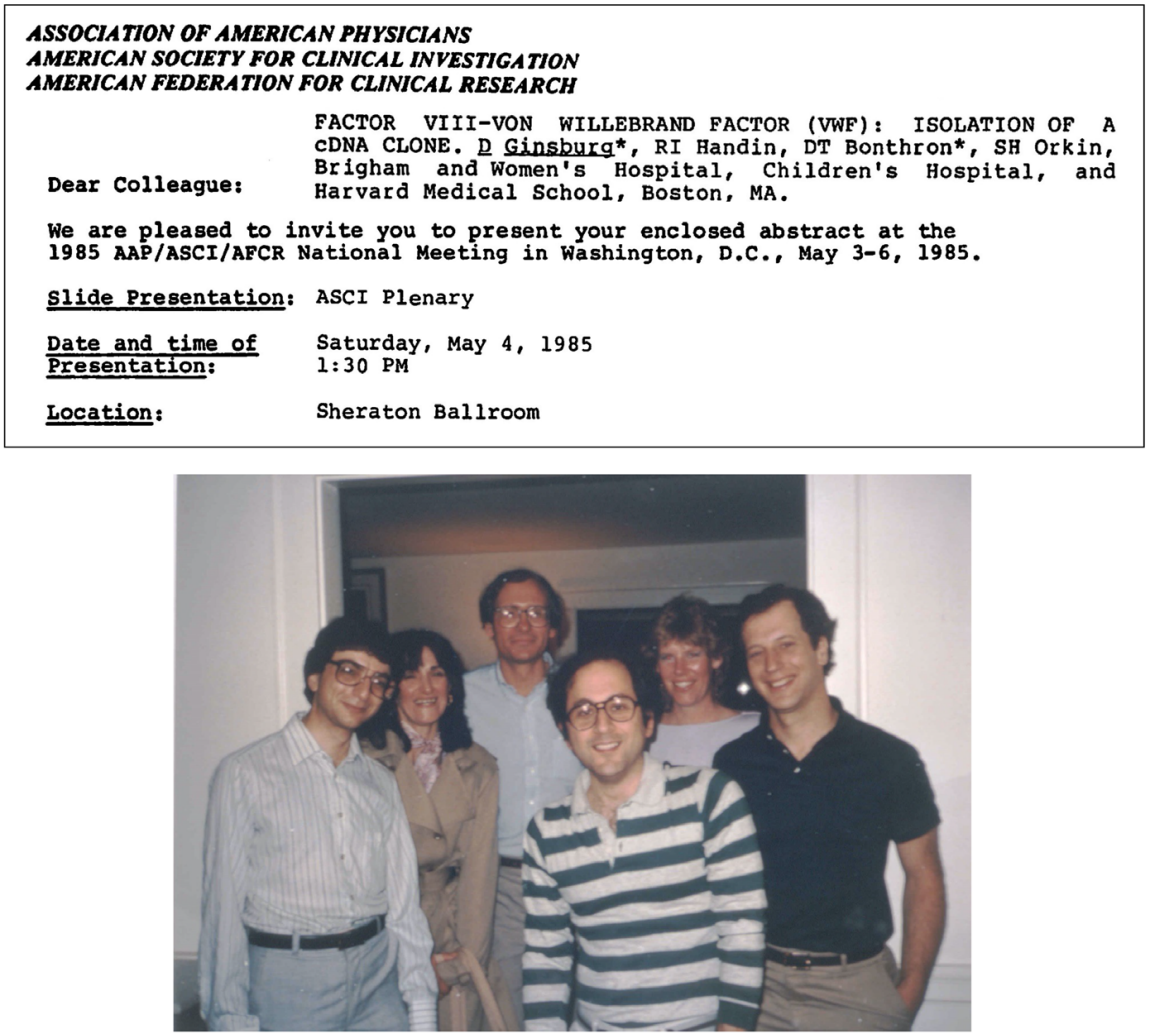
Early career influences. Top panel: The paper notice (by mail) of the selection of my abstract for presentation at the 1985 Clinical Meetings — the predecessor of today’s annual AAP/ASCI/APSA Joint Meeting. Bottom panel: Stuart Orkin’s lab circa 1985, with me first from the right and Stu, third from the right.

I am profoundly honored to have been selected to deliver the 2024 Kober Lecture of the Association of American Physicians (AAP). I attended my first ASCI/AAP meeting (then the Tri-Societies meeting) in 1985, when I was a postdoctoral fellow in Stu Orkin’s lab in Boston. In those days, cDNA cloning was the hot new cutting-edge technology, and under Stu’s direction, I’d succeeded in cloning the cDNA for von Willebrand factor (VWF), and our Abstract on this work was selected for the ASCI Plenary Session (Figure 1). That meeting had a profound influence on me, particularly the opportunity to interact with the leading physician-scientists, my role models, and the giants of science and medicine. I have been attending the meeting regularly ever since.

I am profoundly grateful to my scientific mentors, who greatly influenced and launched my own scientific career. My first mentor was Joan Steitz — I was incredibly lucky to stumble into a position as the second undergraduate student working in her lab, which first introduced me to the joys and rewards of discovery and a career in science. I then returned to science as a hematology fellow, when again by a stroke of incredible luck, Stu Orkin was the attending for my month on the pediatric hematology service and I was fortunate enough to join his lab as his second postdoctoral fellow (Figure 1). Under Stu’s guidance, I combined my interests in genetics and hematology to study VWF and von Willebrand disease (VWD) (1). I think part of what attracted me to study VWD was the similarity to the complexity of the hemoglobin disorders, for which Stu was the pioneer for unraveling at the molecular level. Similarly, for VWD, having the VWF sequence in hand laid the foundation for future work in my laboratory for a number of years dissecting the various subtypes of VWD, which turned out to be due to mutations in different parts of the molecule interfering with specific VWF functions (2–6).

## From bedside to bench

A key privilege of being an academic physician-scientist is the ability to follow one’s passion, to work on whatever excites or interests us at the time. A major theme that I wanted to emphasize in my Kober Lecture was the bedside-to-bench aspect of what we do as physician-scientists. Much attention is given to the pathway from bench to bedside; that is, reducing a basic laboratory finding to a treatment or intervention of value for patients. But another area that we are very privileged to leverage as physician-scientists is in the opposite direction, going from the bedside to the bench; that is, taking observations from our patients and bringing them back to the lab to ask, and potentially answer, fundamental basic scientific questions.

The first paper from my independent laboratory at the University of Michigan reported the cDNA cloning of another blood clotting protein, plasminogen activator inhibitor-1 (PAI-1) (7), subsequently identifying a patient with a rare bleeding disorder due to mutations in this gene (8). With the advent of positional cloning in the 1990s, we went on to identify several other disease genes responsible for rare bleeding and thrombotic disorders, including familial thrombotic thrombocytopenic purpura (TTP), due to loss-of-function mutations in the gene encoding the metalloprotease ADAMTS13 (9). This enzyme is a critical regulator of VWF function, and another example of starting at the bedside in patients with this puzzling rare genetic disorder, with the genetics then leading us to the *ADAMTS13* gene and new insight into the molecular mechanisms underlying TTP. In this example, the basic laboratory bench work identifying this gene laid the foundation for the identification of genetic mutations in families with familial TTP, thus taking this work from the bench back to the bedside, facilitating genetic diagnosis. In addition, expression of recombinant ADAMTS13 led to the production of a new therapeutic for patients with TTP (recently approved by the FDA).

## Combined deficiency of factor V and factor VIII

**Figure 2:**
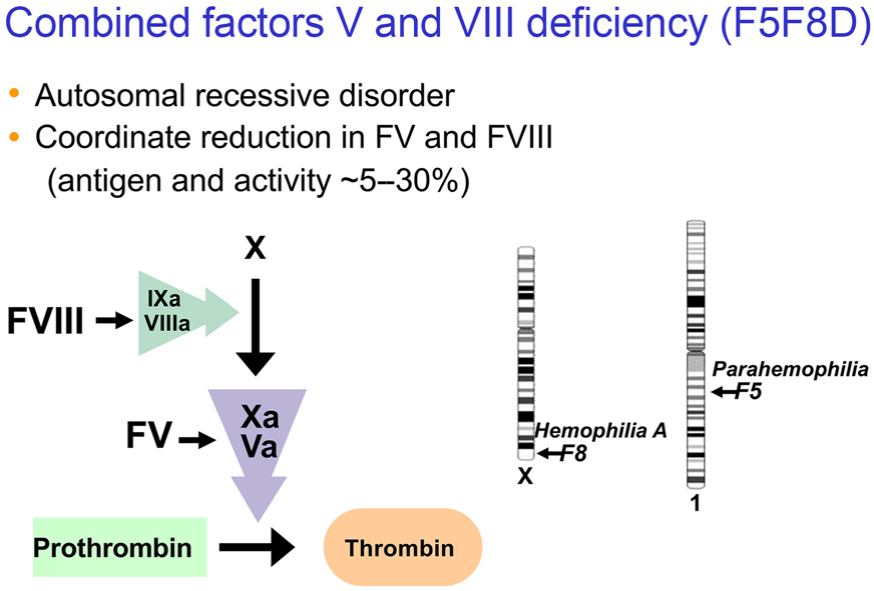
Description of the clinical features of F5F8D, and depiction of FV and FVIII function (the FVIIIa/FIXa complex activates FX to FXa, with the FXa/FVa complex activating prothrombin to thrombin). The location of the *F5* and *F8* genes are depicted on chromosome 1 and the X chromosome, respectively.

For the remainder of this article, I will focus on work from my laboratory over the past 20 years that began with the study of another very rare inherited human bleeding disorder, combined deficiency of coagulation factor V (FV) and FVIII (F5F8D). This fascinating disorder presented an intriguing puzzle for a physician-scientist (Figure 2). F5F8D is an autosomal recessive disorder, which we thus knew must be due to mutations in a single gene. However, the *F8* gene is located on the X chromosome, with its isolated deficiency resulting in the X-linked recessive disorder, hemophilia A, and the *F5* gene is located on chromosome 1, with its isolated deficiency resulting in autosomal recessive parahemophilia. In addition, FV and FVIII are produced in different cell types, with levels in blood that differ by approximately 100-fold. Yet, mutations in this single F5F8D gene somehow simultaneously reduce the levels of both proteins to approximately 10% of normal. We again applied the now “historic” positional cloning approach to DNA obtained from multiple F5F8D families to track down the location of 2 different genes (10, 11). The first gene, previously known as *ERGIC53* (for endoplasmic reticulum [ER]/Golgi intermediate compartment 53 kDa protein), now referred to as *LMAN1*, accounts for approximately 70% of patients. The second gene, *MCFD2* (for multiple coagulation factor deficiency number 2 gene), accounts for the remaining 30%.

**Figure 3:**
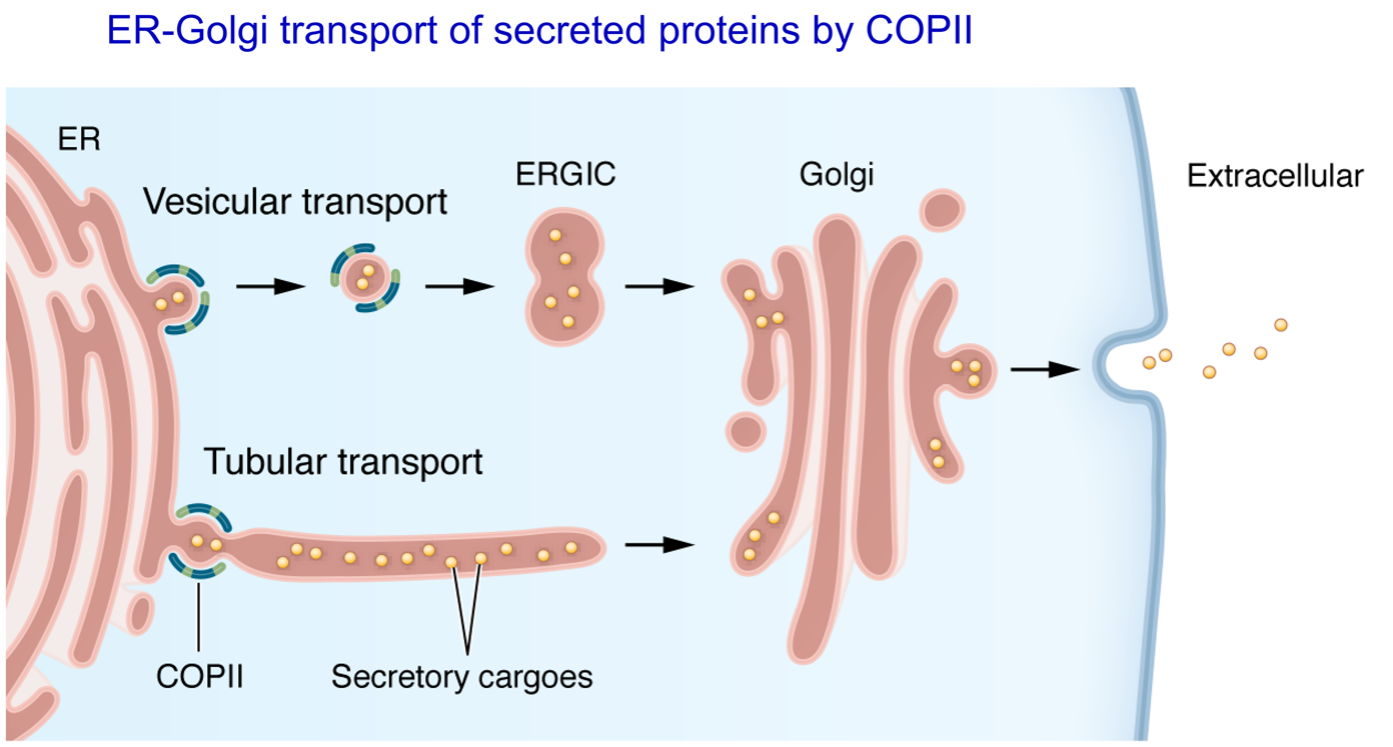
Secretory proteins in the ER lumen are recruited into the COPII vesicle/tubule by COPII coat proteins. In the vesicular transport model, the vesicle buds from the ER and travels to the ERGIC/*cis*-Golgi network with COPII coat proteins accompanying the vesicle. In the tubular transport model, cargo proteins are transported in a continuous interwoven tubular network instead of discrete vesicles. COPII proteins remain on the ER membrane and function as a gatekeeper restricting entry of secretory proteins into tubules. Reproduced from Tang and Ginsburg (12).

We subsequently showed that these two proteins form a complex in the ER, which serves as a cargo receptor for FV, FVIII, and several other proteins. This fascinating finding, another example of bedside-to-bench research, brought my research group into the very basic laboratory bench study of protein transport from the ER to the Golgi (Figure 3) (12). Approximately 20%–30% of proteins encoded by the mammalian genome are dependent on transport through the ER-Golgi secretory pathway, subsequently secreted from the cell, anchored in the cell membrane, or redirected to the lysosome or other intracellular organelle (12). Transport from the ER to Golgi is mediated by the COPII machinery, as so elegantly demonstrated by the pioneering studies of Randy Schekman and others working in the yeast system. This was an entirely new field for my laboratory team and we were aided and learned an enormous amount through collaboration with the Schekman lab and others.

## Mammalian COPII components

**Figure 4:**
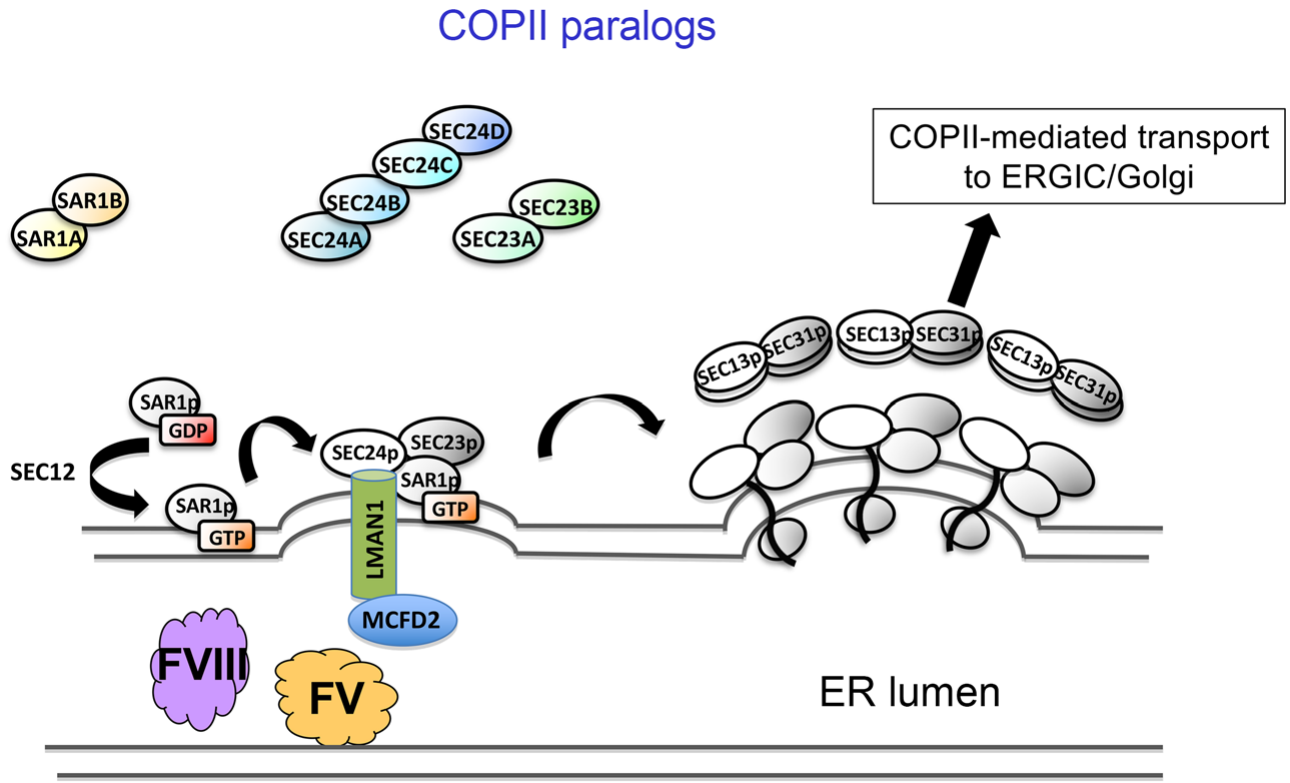
The assembly of the COPII inner and outer coats. The multiple mammalian SAR1, SEC23, and SEC24 paralogs are shown in yellow, green, and blue, respectively. The LMAN1/MCFD2 cargo receptor is shown, interacting with FV/FVIII within the ER lumen and SEC24 on the cytoplasmic face of the ER.

Early in this process, SAR1 GTPase recruits the SEC23/SEC24 heterodimer to the cytoplasmic face of the ER, forming the inner coat of COPII, with SEC13/SEC31 forming the COPII outer coat (reviewed in ref. 12). In mammals there are two paralogs for SAR1, two for SEC23, and four paralogs for SEC24 (Figure 4), raising the intriguing question of why do humans and other mammals have all these extra copies of these genes and what do they do? It was known that transmembrane secreted proteins can interact through their cytoplasmic tails with the SEC24 component of the COPII coat. However, how soluble proteins restricted to the ER lumen are recruited into the COPII vesicle/tubule for transport out of the ER remains an important question. Competing models for this process include bulk flow, in which secreted ER luminal proteins make their way passively into the COPII vesicle/tubule, and receptor-mediated transport, in which a specific transmembrane ER cargo receptor binds the cargo protein within the ER lumen and interacts with the COPII coat on the cytoplasmic face of the ER (Figure 4).

Our findings had identified the LMAN1/MCFD2 complex as such a cargo receptor, interacting with the COPII coat on the cytoplasmic face of the ER and with FV and FVIII within the ER lumen. Although work from our lab and others identified a limited number of other potential cargoes for LMAN1/MCFD2 (reviewed in ref. 12), information about the broader cargo repertoire for this cargo receptor, as well as the identity of other potential ER cargo receptors, remained important questions. Other intriguing questions in this area included the potential functions of the multiple vertebrate paralogs for SEC23, SEC24, and SAR1.

Around this time, a powerful new resource for the study of gene function in mice had been developed (13), which included a large collection of mouse embryonic stem (ES) cells carrying inactivated alleles for many/most genes in the mouse genome. Surveying this database in the mid 1990s, I was surprised to find knockout ES cells available for nearly all of the COPII coat components. I couldn’t resist the temptation, so our lab went on to develop knockout mice for *Sar1a* (14), *Sar1b* (14), *Sec23a* (15), *Sec23b* (16), and all 4 *Sec24* (*a*–*d*) paralogs (17–19). Only a few human diseases are known to be due to mutations in the human homologs for these genes. However, the range of phenotypes observed in mice with homozygous deficiency for each of these genes was remarkably different from what had been reported in humans (Table 1).

*SEC23A* deficiency in humans results in a unique skeletal abnormality, whereas the corresponding deficiency in mice is lethal during mid embryogenesis. *SEC23B* deficiency in humans results in the unique red cell disorder congenital dyserythropoietic anemia type II (CDAII). In contrast, mice with complete deficiency for *Sec23b* develop a severe exocrine pancreas defect and die shortly after birth. What accounts for these remarkably different pathologies in humans and mice? The explanation appears to lie in an evolutionary shift in the gene expression programs for these 2 paralogous genes. *SEC23B* is the major SEC23 paralog expressed in human red blood cell precursors, with higher levels of *Sec23a* expressed in the corresponding mouse cells. Similarly, *Sec23b* is the predominant paralog in mouse pancreas, with considerably higher *SEC23A* expression in human pancreas. These data suggested that differences in the gene expression programs could explain the different mouse and human phenotypes, rather than actual differences in function between the SEC23A and SEC23B proteins. A very talented physician-scientist postdoc in the lab, Rami Khoriaty (now directing his own highly successful independent laboratory), generated a mouse model in which the SEC23A coding sequence was substituted for that of SEC23B at the endogenous *Sec23b* gene locus (20). These mice thus completely lack the SEC23B protein, but express the SEC23A protein in all the places where the *Sec23a* or *Sec23b* genes would normally be expressed. This genetically engineered mouse showed complete rescue of the lethal pancreatic phenotype, with mice surviving to adulthood and appearing entirely normal. These findings thus demonstrate complete or near-complete overlap in function between SEC23A and SEC23B in vivo.

We recently published a similar analysis for the mouse *Sar1a* and *Sar1b* genes (14). *SAR1B* deficiency in humans results in a unique lipid disorder termed Anderson disease or chylomicron retention disease (CMRD). Although homozygous *Sar1b* deficiency in mice results in early embryonic lethality, replacement of the murine SAR1B coding sequence with that of SAR1A resulted in full rescue of the lethal murine *Sar1b*-null phenotype, again demonstrating complete or near-complete overlap in function between these two closely related paralogs.

Distinct among mice with complete deficiency of a COPII protein (most of whom exhibit embryonic or perinatal lethality, Table 1) were mice with homozygous deficiency for *Sec24a* (19). The latter mice appeared grossly normal, with normal survival. A trainee in the lab misinterpreted a previous paper from the Brown and Goldstein lab and measured cholesterol levels in these mice. After explaining to him why this was an error and not worth measuring, we were surprised to learn that in fact, *Sec24a*-null mice exhibit significantly reduced cholesterol levels (about half of normal controls). Following up on this observation, a very talented postdoc, Xiao-wei Chen (who now runs his own highly productive laboratory at Peking University in Beijing) went on to solve this puzzle (19). Xiao-wei observed that plasma PCSK9 levels are significantly reduced in *Sec24a*-null mice (to approximately half of normal controls). Previous landmark work from an outstanding physician-scientist and great friend and colleague (and previous Kober Lecturer), Helen Hobbs, had identified PCSK9 as a key regulator of the LDL receptor. PCSK9 circulates in blood and when bound to the LDL receptor mediates its trafficking to the lysosome and subsequent degradation. This mechanism explains why patients with reduced PCSK9 levels have increased LDL receptor on the hepatocyte cell membrane, resulting in increased LDL clearance and reduce plasma cholesterol levels. Helen’s remarkable discovery, a terrific example of bedside-to-bench and back again research, opened up an entirely new field and led to the development of anti-PCSK9 antibodies and other strategies as therapeutic approaches for the treatment of hypercholesterolemia.

Xiao-wei showed that the PCSK9 levels in the blood of *Sec24a*-null mice are approximately half those of normal controls, with no difference in hepatic mRNA levels. These findings raised a fascinating question. PCSK9 is a soluble protein restricted entirely to the ER lumen on its way through the secretory pathway, with SEC24 restricted entirely to the cytoplasmic face of the ER membrane (Figure 4). Thus, how could PCSK9 know that it was SEC24A on the other side of the ER membrane and not SEC24B, -C, or -D? This observation suggested that there must be a cargo receptor that spans the ER membrane and links PCSK9 on the ER luminal side to SEC24A on the cytoplasmic side. Very few cargo receptors had been defined in mammalian systems, with LMAN1/MCFD2, the FV and FVIII cargo receptor, being one of the few well-characterized such cargo receptors. However, patients with F5F8D are not known to exhibit reduced cholesterol levels, suggesting that there must be another cargo receptor for PCSK9.

## Another mammalian cargo receptor

To identify this putative cargo receptor, another very talented physician-scientist trainee in the lab, Brian Emmer (also now directing his own highly productive independent lab at the University of Michigan), decided to take on this problem. Brian developed a unique assay to measure PCSK9 secretion by tagging it with green fluorescent protein (GFP). His idea was that in a reporter cell expressing this PCSK9-GFP fusion protein, inactivation of a gene critical for expression of the putative PCSK9 cargo receptor would result in increased intracellular accumulation of GFP. Using such a reporter cell line, Brian performed a whole-genome CRISPR screen using the library and strategy developed by the Zhang lab at MIT (21). In this experiment, cells expressing this PCSK9-GFP fusion were infected with a lentiviral library containing more than 120,000 unique guide RNAs (~6 for each gene in the genome). Each infected cell will thus have a different gene inactivated, depending on the guide RNA it received. Cells receiving a guide RNA targeting the PCSK9 cargo receptor, or some other key component of the secretory pathway unique for PCSK9, would accumulate green fluorescence (with a second control secretory protein labeled in red). Brian then sorted cells for a normal level of control protein (red) fluorescence, but accumulation of GFP-tagged PCSK9, with the selected cells then subject to next-generation sequencing to identify the enriched guide RNAs (corresponding to those genes/proteins that when inactivated lead to a block in PCSK9 secretion).

This experiment resulted in one very clear and dramatic “hit”— out of all the genes in the genome, there was only one that had a major effect on PCSK9 intracellular accumulation (22). Indeed, the top four guide RNAs that gave the strongest signals (out of the ~123,000 guides in the library) were four of the six guide RNAs for the same gene, a gene known as *SURF4*. *SURF4* encodes a protein that was known to localize to the ER and ERGIC compartments and also exhibits homology to a well-characterized ER cargo receptor in yeast, Erv29p. The ortholog of *SURF4* in *C*. *elegans*, *sft-4*, had previously been shown to mediate secretion of the yolk protein, VIT-2 (23). This latter paper demonstrated that SURF4 is also required for efficient secretion of another mammalian protein, ApoB, in HepG2 cells. Subsequent work from a number of other labs has identified several other potential cargoes for SURF4 (24–26). Although we showed that germline deletion of *Surf4* results in early embryonic lethality in mice (27), mice with SURF4 deficiency restricted to hepatocytes survive and appear essentially normal, except for an approximately 50% reduction in PCSK9 levels in plasma and a remarkable reduction in plasma cholesterol to approximately 10% of normal, likely due to an effect on other cholesterol-related proteins, including ApoB, which is markedly reduced in the plasma of hepatocyte-specific *Surf4*-knockout mice (28).

## Cargo receptor repertoires

The findings from our studies of these two cargo receptors, SURF4 and LMAN1/MCFD2, raised an interesting question as to what other secreted proteins might be dependent on each of these cargo receptors. To address this question in an unbiased way, we developed a proteomics approach for analysis of the whole-cell secretome. For these studies, cells are grown in culture, quantitative mass spectrometry analysis performed on the conditioned media and cell lysates, and the quantitative ratio for cell media to lysate calculated for each protein. This approach controls for abundant cytoplasmic proteins that are released into the media from sick or dying cells. Indeed, the proteins with high media/lysate ratios were dramatically enriched for known secretory proteins and depleted for known intracellular proteins (29). We recently performed this analysis on HuH7 hepatoma cells that had been genetically engineered to inactivate either *LMAN1* or *SURF4*. Media/lysate ratios from the *LMAN1*- and *SURF4*-knockout cells compared with wild-type controls should identify the full range of potential cargoes for each of these cargo receptors. Surprisingly, analysis of *LMAN1*-knockout cells identified a very limited set of potential cargoes. In contrast, analysis of *SURF4*-deleted cells identified a large collection of potential SURF4 cargoes, including ApoB (30). Taken together, these findings suggest that LMAN1/MCFD2 may be highly specific, with a very limited cargo repertoire, whereas SURF4 may have a much wider repertoire, with evidence suggesting that it may be particularly important for abundantly expressed proteins.

## The privilege of being a physician-scientist

In conclusion, what I’ve tried to emphasize in this lecture is the remarkable role we can play as physician-scientists, making fundamental observations at the patient bedside and taking them all the way to the very basic laboratory, including such fundamental cellular processes as COPII-mediated protein transport. These basic studies also provide numerous opportunities for going back from the bench to the bedside. From the work I have reviewed here, a number of possibilities come to mind, including the potential targeting of SEC24A or SURF4 for the treatment of hypercholesterolemia, the targeting of LMAN1/MCFD2 as a potential anticoagulant approach, and strategies to elevate expression of overlapping genetic paralogs to treat diseases due to genetic deficiency in another paralog. For example, increasing the expression of SEC23A could be considered to treat congenital CDAII (due to deficiency of SEC23B), or SAR1A to treat Anderson disease (due to deficiency of SAR1B). These latter examples would be analogous to the highly successful approach of increasing human fetal hemoglobin expression in adults with sickle cell anemia to compensate for an abnormal β-globin gene, the first FDA-approved CRISPR therapy, based on the work of my wonderful mentor and friend, Stuart Orkin (31).

Finally, analogous to the cycle of taking basic observations at the patient bedside to the laboratory bench and then back again from the bench to the bedside, another enormous privilege we have as academic physician-scientists is the opportunity to participate in the virtuous cycle of mentoring. I feel profoundly indebted to my wonderful scientific mentors who introduced me to science and prepared me for my own very rewarding career, but I am also enormously grateful for the opportunity this career has given me to pay it forward with the mentoring of my own students and trainees whose aggregate scientific contributions will certainly far exceed my own.

It was a fantastic privilege to be selected to present the 2024 Kober Lecture, and I am deeply humbled to be added to the list of Kober Lecturers, which includes the names of many of the physician-scientists that I have most admired throughout my career. I am very indebted to the AAP and the ASCI for the wonderful role both Societies have played, and continue to play, in my own career and I’m also very grateful to the NIH for its support throughout my career and to the Howard Hughes Medical Institute for supporting me from the very beginning of my independent career in 1985 until my phase out of the Investigator Program in September of 2023, though I remain associated as an emeritus HHMI Investigator.

## Figures and Tables

**Table 1:**
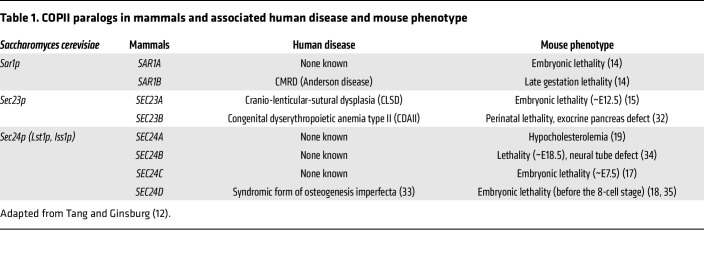
COPII paralogs in mammals and associated human disease and mouse phenotype

## Version 1 11/15/2024

Electronic publication
